# Reassortant Highly Pathogenic Influenza A H5N2 Virus Containing Gene Segments Related to Eurasian H5N8 in British Columbia, Canada, 2014

**DOI:** 10.1038/srep09484

**Published:** 2015-03-25

**Authors:** John Pasick, Yohannes Berhane, Tomy Joseph, Victoria Bowes, Tamiko Hisanaga, Katherine Handel, Soren Alexandersen

**Affiliations:** 1 Canadian Food Inspection Agency, National Centre for Foreign Animal Disease, Winnipeg, Manitoba, Canada R3E 3M4; 2 Animal Health Centre, Ministry of Agriculture, Abbotsford, British Columbia, Canada V3G 2M3

## Abstract

In late November 2014 higher than normal death losses in a meat turkey and chicken broiler breeder farm in the Fraser Valley of British Columbia initiated a diagnostic investigation that led to the discovery of a novel reassortant highly pathogenic avian influenza (HPAI) H5N2 virus. This virus, composed of 5 gene segments (PB2, PA, HA, M and NS) related to Eurasian HPAI H5N8 and the remaining gene segments (PB1, NP and NA) related to North American lineage waterfowl viruses, represents the first HPAI outbreak in North American poultry due to a virus with Eurasian lineage genes. Since its first appearance in Korea in January 2014, HPAI H5N8 spread to Western Europe in November 2014. These European outbreaks happened to temporally coincide with migratory waterfowl movements. The fact that the British Columbia outbreaks also occurred at a time associated with increased migratory waterfowl activity along with reports by the USA of a wholly Eurasian H5N8 virus detected in wild birds in Washington State, strongly suggest that migratory waterfowl were responsible for bringing Eurasian H5N8 to North America where it subsequently reassorted with indigenous viruses.

Since 2003 highly pathogenic avian influenza (HPAI) Eurasian H5N1 viruses of the A/goose/Guandong/1/1996 lineage (Gs/GD-lineage) have become endemic in poultry in Bangladesh, China, India, Indonesia, Vietnam, and Egypt^1^. Trade of poultry and poultry products as well as the movements of infected wild birds have been implicated in this spread^2^ and concern that migratory birds could introduce this virus to the Americas resulted in enhanced surveillance for H5N1 in wild birds in Canada and the United States^3^,^4^,^5^,^6^. Over this same time period there have been 668 confirmed human cases and 393 deaths due to H5N1 causing concern that H5N1 represents a real zoonotic and potential pandemic threat to humans. Furthermore, Gs/GD-lineage H5N1 viruses have undergone extensive evolution giving rise to 10 clades (0–9) with some of those clades containing subclades (http://www.who.int/influenza/gisrs_laboratory/201101_h5fulltree.pdf). Despite extensive reassortment that has resulted in the incorporation of non-H5N1 internal gene segments, the NA gene of these viruses remained Gs/GD-like until recently. Novel HPAI H5N2, H5N5, H5N6 and H5N8 viruses began appearing in China in 2009^7^,^8^,^9^ and in 2014 novel HPAI H5N8 viruses were isolated from poultry and wild waterfowl in South Korea^10^,^11^. Genetic analyses of the viruses isolated from poultry farms and wild birds during the initial phase of the South Korean outbreaks showed that they were identical to one another with both belonging to the proposed clade 2.3.4.6^12^. More recently, after reviewing the available H5 sequence data, the WHO/OIE/FAO H5N1 Evolution Working Group has decided that the current H5N6 and H5N8 viruses be designated as clade 2.3.4.4 (http://www.who.int/influenza/gisrs_laboratory/h5_nomenclature_clade2344/en/). Here we report the first HPAI outbreak in North America that involves a virus with a Eurasian Gs/GD-lineage HA gene.

The genetic relatedness of the HPAI H5N8 viruses isolated from poultry and wild waterfowl coupled with an increase in the H5 seroprevalence and detection of HPAIV in wild birds in 2014 suggested that migratory birds may have played a key role in introducing and spreading this virus during the initial stage of the South Korean 2014 outbreak. Between November 5 and December 16, 2014 outbreaks of HPAI Eurasian H5N8 in poultry were reported in the Netherlands, Germany, the United Kingdom and Italy^13^,^14^. In all four outbreaks the responsible viruses were phylogenetically related to the South Korean H5N8 viruses^14^. Additionally, the absence of epidemiological evidence linking the outbreaks in Germany, the Netherlands and the United Kingdom coupled with the fact that H5N8 HPAIV was detected in wild bird populations in Germany and the Netherlands^15^, and that in all three outbreaks there was proximity to areas with wild birds, suggests that wild migratory birds may have been a possible source of the virus. The temporal association of all the European outbreaks also happened to have coincided with the movements of migratory waterfowl.

## Results and Discussion

**Figure 1:**
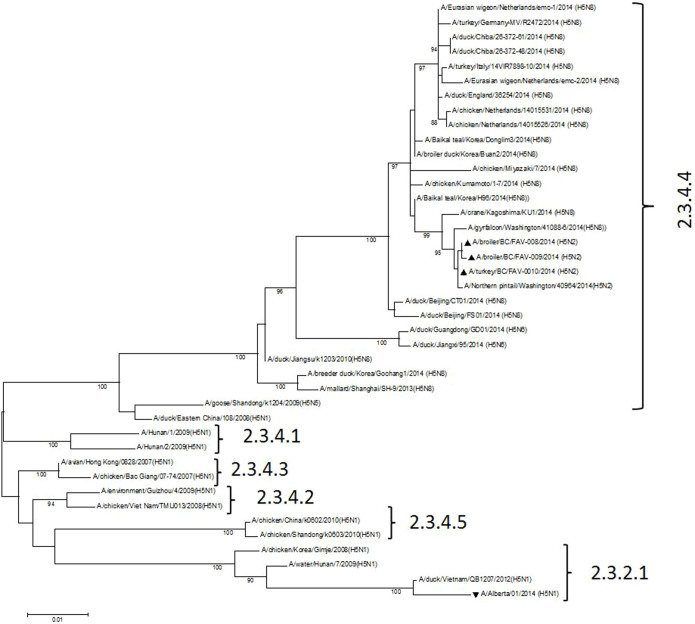
Phylogenetic characterization of the hemagglutinin genes of Canadian HPAI H5N2 virus isolates. The complete hemagglutinin (HA) gene sequences of the Canadian HPAI H5N2 viruses were aligned with H5 genes obtained from NCBI and GISAID EpiFlu™ databases using Clustal W. The supplementary file contains additional phylogenetic trees for the remaining gene segments and a Table that list and acknowledges the sequences included from GISAID. Phylogenetic and molecular evolutionary analysis was conducted using MEGA version 6 and the Maximum Likelihood method based on the Tamura Nei model^28^. The tree with the highest log likelihood is shown. Bootstrap test involved 500 replicates to determine reliability of the inferred tree with only bootstrap values above 70% shown. Black upright triangles, Canadian HPAI H5N2; inverted triangle single Canadian human case of HPAI H5N1. Clade designations are based on WHO/OIE/FAO H5N1 Evolution Working Group nomenclature (http://www.who.int/influenza/gisrs_laboratory/h5_nomenclature_clade2344/en/).

On November 28, 2014, in Abbotsford, British Columbia, Canada a sudden increase in mortality was observed in a flock of 11,000 83-day old meat turkeys. The mortality rate would exceed 70% over the next 3 days. On November 30, 2014 a broiler breeder flock 8 km away in neighbouring Chilliwack, British Columbia experienced a10% mortality over a 24 hr period. Post-mortem examination of the chickens revealed lesions compatible with HPAI. These included severe facial edema, conjunctivitis, tracheal hyperemia, severe pulmonary congestion and edema and hemorrhages involving the mucosa of the proventriculus. Shank (tarsal) hemorrhages were also present. In contrast, the lesions in the turkeys were limited to varying degrees of generalized dark patchy congestion of the skeletal musculature and mild superficial congestion of the cecal tonsils. In both cases RNA extracted from tracheal swabs, cloacal swabs and tissues were positive for H5 subtype virus by real-time reverse transcription PCR. Using a universal primer set^16^ the full sequence of all 8 genes was obtained directly from diagnostic specimens and viral isolates from these two outbreaks. Based on BLAST searches (http://blast.ncbi.nlm.nih.gov/Blast.cgi) the closest gene sequences of the 8 viral gene segments in the NCBI database (Table 1) were identified which suggested the presence of a novel reassortant HPAI H5N2 virus that possessed gene segments related to Eurasian HPAI H5N8 viruses and North American lineage waterfowl viruses. The GenBank accession numbers for the HPAI H5N2 turkey isolate and the broiler isolates are KP307954 - KP307961 and KP795726 - KP795741. Phylogenetic analysis of the HA inferred a higher degree of relatedness to 2014 Korean H5N8 rather than 2014 UK, German and Dutch H5N8 isolates (Figure 1). It is important to note that the HA of the Canadian poultry H5N2 viruses was unrelated to the H5N1 that was associated with the human case in Alberta, Canada in 2013^17^. Phylogenetic trees for the remaining gene segments are found in supplementary Figures 1 through 7 that also contains a Table which list and acknowledge the sequences included from GISAID. Viruses isolated from the chicken broiler breeder and meat turkey farms were essentially genetically identical and had intravenous pathogenicity indices of 2.98 and 2.96 respectively. The deduced amino acid sequence of the connecting peptide of the HA is LRERRRKR/GLF and identical to that reported for 2014 Korean HPAI H5N8 viruses. Amino acids E190, R220, G225, Q226 and G228 (H3 numbering) involved in receptor binding indicates that this virus has an affinity for α-2,3-linked glycans^18^ or avian specificity, however, amino acids A138 and A160, also present in this virus, have been previously reported to be associated with enhanced binding to α-2,6-linked glycans or human receptor specificity^19^. The NA, of typical North American wild bird origin, shows no stalk truncation which has been associated with adaptation to gallinaceous poultry^20^ suggesting a very recent introduction of this virus into poultry. The NA catalytic site residues R118, E119, D151, R152, W178, I222, R224, E227, A246, E276, R292, and R371 along with H274 indicate susceptibility to antiviral neuraminidase inhibitors, however the asparagine 31 mutation present in the M2 protein is associated with resistance to amantadine^21^. The virus has a glutamic acid instead of a lysine at amino acid residue 627 of the PB2 protein further indicating an avian adapted virus. In summary, this represents the first HPAI outbreak in North America that involves a virus with a Eurasian Gs/GD-lineage HA gene. Some of the viruses, such as H5N1 and H5N6, bearing the HA gene of this lineage have a proven zoonotic and possibly pandemic potential and for this reason a risk analysis for potential human infection should be carried out, the situation followed very closely and everything done to decisively control and eradicate the infection in poultry.

Based on limited information at the time, we speculated that migratory waterfowl were responsible for the movement of Eurasian HPAIV H5N8 from Asia to North America where it subsequently reassorted with North American lineage wild bird viruses. This hypothesis was very quickly supported by the finding of a wholly Eurasian H5N8 virus in a gyrfalcon (*Falco rusticolus*) and a Eurasian/North American reassortant H5N2 virus, similar to the one causing outbreaks in British Columbia, in a northern pintail (*Anas acta*)^22^. Both were found in association with wild bird mortalities in Wiser Lake in Whatcom County, Washington State, USA, 40 km from the Canadian outbreak nidus.

The first indication that wild birds could spread Eurasian H5N1 avian influenza involved outbreaks in wild waterfowl and captive wild birds in Hong Kong in late 2002^23^. However, it was not until the large outbreak involving wild birds on Lake Qinghai in May 2005^24^ that concern over the involvement of wild waterfowl in the spread of H5N1 avian influenza came to the forefront. Of the 23 introductions of H5N1 avian influenza in Europe, 20 were associated with migrating wild birds^25^. Nevertheless, evidence for the intercontinental movement of avian influenza viruses from Asia to North America in migratory birds has been sparse. Northern pintails, which is one of the most abundant duck species at high latitudes and has been shown by banding and radio-transmitter studies to occasionally have direct migratory movements between Asia and North America, were the subject of a study looking at the intercontinental movement of avian influenza viruses^26^. Whole genome analysis carried out on 38 LPAIV isolated from northern pintails in Alaska in 2006 showed that 44.7% of the isolates had evidence for reassortant events with viruses of Asian lineage^26^. The authors suggested that species like northern pintails which maintain a relatively high prevalence of influenza viruses, migrate long distances and are potentially exposed to a diversity of avian influenza virus gene pools, have a greater chance of virus co-infection and hence reassortment. Further transmission may then potentially occur to other bird species with the risk of additional migratory spread by abundant waterfowl such as mallards, teals, swans and geese as well as mortality in raptors feeding on such birds. Should this reassortant H5N2 and/or the Eurasian H5N8 ancestor manage to establish or already have established its self in the migratory or resident wild bird population in North America, the long term significance of this may be immense and unpredictable.

This event involving the introduction of the Eurasian H5 lineage of HPAI virus is likely to be of historical significance and raise the need for increased surveillance activities directed towards detecting intercontinental movement of influenza A viruses in wild birds.

## Methods

RNA was extracted from swab specimens or tissue homogenates using a MagMAX™-96 AI/ND Viral Extraction Kit (Ambion) and a MagMAX Express 96 particle processor. The 8 influenza A virus gene segments were amplified in a one-step RT-PCR using a universal primer set^16^ and a high-fidelity RT-PCR kit (Invitrogen; Superscript III One-Step RT-PCR System with Platinum TaqHigh Fidelity). RT-PCR amplicons were cloned into pCR4-Topo (Invitrogen) and used to transform OneShot TOP10 or Mach™-T competent *E. coli*. The resulting plasmids were sequenced using BigDye Terminator chemistry version 3.1 (Life Technologies) and an Applied Biosystems 3130xl Genetic Analyzer. A consensus sequence for each gene segment was arrived at based on the results of bidirectional sequencing of a minimum of 4 clones. Representative reference sequences with Eurasian and North American geographical associations were selected from NCBI and GISAID databases and aligned with the HA of the British Columbia H5N2 virus isolates using Clustal W. Phylogenetic analysis was conducted in MEGA6^27^ using the maximum likelihood method based on the Tamura-Nei model^28^. The initial trees for the heuristic search were obtained by applying Neighbor-Joining and BioNJ algorithms to a matrix of pairwise distances estimated using the maximum composite likelihood approach. Five-hundred bootstrap replicates were used to determine the reliability of the inferred tree.

All animal experiments were performed in strict accordance with the recommendations in the Canadian code of practice for the care and use of animals and was approved by the National Centre for Foreign Animal Disease (NCFAD) Animal Care Committee.

The intravenous pathogenicity indices (IVPI) of the virus isolates were determined as described in the World Organization for Animal Health (OIE) Manual of Diagnostic Tests and Vaccines for Terrestrial Animals^29^. Briefly, a total of 10 specific pathogen free (SPF), 4- to 6-week-old White Leghorn chickens (*Gallus gallus domesticus*) were inoculated intravenously with 0.1 ml of each virus stock that had been diluted 1:10 in sterile PBS. The hemagglutination (HA) titer of the H5N2 broiler chicken isolate used in the IVPI was 1024 while the HA titer of the H5N2 turkey isolate was 32. Ten control birds were inoculated with 0.1 ml of PBS by the intravenous route. Birds were observed daily for a total of 10 days and given a clinical score at the end of each 24 hr period^29^. The IVPI is the mean score per bird per observation period over the 10 day period. A maximum score of 3.00 means that all birds died by 24 hours post-inoculation. Viruses with IVPI > 1.2 are considered highly pathogenic.

## Author Contributions

T.J. and V.B. coordinated the work done at the BC Animal Health Centre while J.P., Y.B. and S.A. coordinated work done at the National Centre for Foreign Animal Disease. T.H. and K.H. performed the nucleic acid sequencing. J.P. drafted the initial manuscript and later versions were based on input and suggestions from S.A. All authors contributed to the final submitted version.

## Supplementary Material

Supplementary Information Supplementary Material

## Figures and Tables

**Table 1:** Nucleotide homology of genes of influenza virus strain A/turkey/British Columbia/FAV10/2014 (H5N2) to the closest influenza virus strains in GenBank

Gene	Closest related virus strain	GenBank accession no.	Nucleotide identity, %	Lineage
**PB2**	A/broiler duck/Korea/H49/2014 (H5N8)	KJ508953.1	2268/2280, 99.5%	Eurasian
**PB1**	A/bufflehead/California/3118/2001 (H4N8)	CY134341.1	2252/2277, 99.0%	North American
**PA**	A/common teal/Korea/H455-30/2014 (H5N8)	KJ509155.1	2111/2123, 99.4%	Eurasian
**HA**	A/Baikal teal/Korea/H96/2014 (H5N8)	KJ509036.1	1691/1704, 99.2%	Eurasian
**NP**	A/American green-winged teal/Ohio/13OS2084/2013 (H6N8)	KJ568114.1	1483/1497, 99.0%	North American
**NA**	A/northern shoveler/California/3769/2012 (H6N2)	CY177031.1	1397/1410, 99.1%	North American
**M**	A/mallard/Korea/W452/2014 (H5N8)	KJ746114.1	985/986, 99.9%	Eurasian
**NS**	A/Baikal teal/Donglim3/2014 (H5N8)	KJ413854.1	853/855, 99.7%	Eurasian

The BLAST analysis was carried out on December 5, 2014.

## References

[b1] Alexander D. J. & Brown I. H. History of highly pathogenic avian influenza. Rev. Sci. Tech. 28, 19–38 (2009). 19618616 10.20506/rst.28.1.1856

[b2] Kilpatrick A. M. *et al.* Predicting the global spread of H5N1 avian influenza. PNAS USA 103, 19368–19373 (2006). 17158217 10.1073/pnas.0609227103 PMC1748232

[b3] Spackman E. *et al.* Characterization of low-pathogenicity H5N1 avian influenza viruses from North America. J. Virol. 81, 11612–11619 (2007). 17728231 10.1128/JVI.01368-07 PMC2168782

[b4] Pasick J. *et al.* Survey of influenza A viruses circulating in wild birds in Canada 2005 to 2007. Avian Dis. 54, 440–445 (2010). 20521675 10.1637/8800-040109-Reg.1

[b5] Pedersen K., Swafford S. R. & DeLiberto T. J. Low pathogenicity avian influenza subtypes isolated from wild birds in the United States, 2006–2008. Avian Dis. 54, 405–410 (2010). 20521670 10.1637/8693-031309-Reg.1

[b6] Reeves A. B. *et al.* Genomic analysis of avian influenza viruses from waterfowl in western Alaska, USA. J. Wildlife Dis. 49, 600–610 (2013). 10.7589/2012-04-108 23778609

[b7] Zhao G. *et al.* Novel reassortant highly pathogenic H5N2 avian influenza viruses in poultry in China. PLoS ONE 7, e46183; 10.1371/journal.pone.0046183 (2012). 23049973 PMC3458027

[b8] Zhao K. *et al.* Characterization of three H5N5 and one H5N8 highly pathogenic avian influenza viruses in China. Vet. Microbiol. 163, 351–357 (2013). 23375651 10.1016/j.vetmic.2012.12.025

[b9] Qi X., Cui L., Yu H., Ge Y. & Tang F. Whole-genome sequence of a reassortant H5N6 avian influenza virus isolated from a live poultry market in China, 2013. Genome A 2, e00706–714 (2014). 25212611 10.1128/genomeA.00706-14 PMC4161740

[b10] Lee Y.-J. *et al.* Novel reassortant influenza A(H5N8) viruses, South Korea, 2014. Emerg. Infect. Dis. 20, 1087–1089 (2014). 24856098 10.3201/eid2006.140233 PMC4036756

[b11] Ku K. B. *et al.* Highly pathogenic avian influenza A(H5N8) virus from waterfowl, South Korea, 2014. Emerg. Infect. Dis. 20, 1587–1588 (2014). 25152954 10.3201/eid2009.140390 PMC4178401

[b12] Jeong J. *et al.* Highly pathogenic avian influenza virus (H5N8) in domestic poultry and its relationship with migratory birds in South Korea during 2014. Vet. Microbiol. 173, 249–257 (2014). 25192767 10.1016/j.vetmic.2014.08.002

[b13] Avian influenza outbreak in Yorkshire: strain identified as H5N8. Vet. Rec. 175, 495–496; 10.1136/vr.g6947 (2014). 25413814

[b14] Adlhoch C. *et al.* Comparing introduction to Europe of highly pathogenic avian influenza viruses A(H5N8) in 2014 and A(H5N1) in 2005. Euro Surveill. 19, 20996 (2014). 25597538 10.2807/1560-7917.es2014.19.50.20996

[b15] Avian influenza: no clear indication of how H5N8 virus entered EU. Vet. Rec. 176, 59; 10.1136/vr.h198 (2015). 25598458

[b16] Hoffman E., Stech J., Guan Y., Webster R. G. & Perez D. R. Universal primer set for the full-length amplification of all influenza A viruses. Arch. Virol. 146, 2275–2289 (2001). 11811679 10.1007/s007050170002

[b17] Pabbaraju K. *et al.* Full-genome analysis of avian influenza A(H5N1) virus from a human, North America, 2013. Emerg. Infect. Dis. 20, 887–891 (2014). 24755439 10.3201/eid2005.140164 PMC4012823

[b18] Stevens J. *et al.* Structure and receptor specificity of the hemagglutinin gene from an H5N1 influenza virus. Science 312, 404–410 (2006). 16543414 10.1126/science.1124513

[b19] Wang W. *et al.* Glycosylation at 158N of the hemagglutinin protein and receptor binding specificity synergistically affect the antigenicity and immunogenicity of a live attenuated H5N1 A/Vietnam/1203/2004 vaccine virus in ferrets. J. Virol. 84, 6570–6577 (2010). 20427525 10.1128/JVI.00221-10 PMC2903256

[b20] Li J., zu Dohna H., Cardona C. J., Miller J. & Carpenter T. Emergence and genetic variation of neuraminidase stalk deletions in avian influenza viruses. PLoS ONE 6, e14722 (2011). 21373190 10.1371/journal.pone.0014722 PMC3044137

[b21] Hay A. J., Wolstenholme A. J., Skehel J. J. & Smith M. H. The molecular basis of the specific anti-influenza action of amantadine. EMBO J. 4, 3021 (1985). 4065098 10.1002/j.1460-2075.1985.tb04038.x PMC554613

[b22] Ip H. S. *et al.* Novel Eurasian highly pathogenic H5 viruses detected in wild birds in Washington State. Emerg. Infect. Dis. Accepted for publication. 10.3201/eid2105.142020 PMC4412248 25898265

[b23] Ellis T. M. *et al.* Investigation of outbreaks of highly pathogenic H5N1 avian influenza in waterfowl and wild birds in Hong Kong in late 2002. Avian Path. 33, 492–505 (2004). 15545029 10.1080/03079450400003601

[b24] Liu J. *et al.* Highly pathogenic H5N1 influenza virus infection in migratory birds. Science 309, 1206 (2005). 16000410 10.1126/science.1115273

[b25] Kilpatrick A. M. *et al.* Predicting the global spread of H5N1 avian influenza. PNAS USA 103, 19368–19373 (2006). 17158217 10.1073/pnas.0609227103 PMC1748232

[b26] Koeher A. V., Pearce J. M., Flint P. L., Franson C. & Ip H. S. Genetic evidence of intercontinental movement of avian influenza in a migratory bird: the northern pintail (*Anas acuta*). Mol. Ecology 17, 4754–4762 (2008). 10.1111/j.1365-294X.2008.03953.x 19140989

[b27] Tamura K., Stecher G., Petersen D., Filipski A. & Kumar S. MEGA6: Molecular evolutionary genetics analysis version 6.0. Mol. Biol. Evol. 30, 2725–2729 (2013). 24132122 10.1093/molbev/mst197 PMC3840312

[b28] Tamura K. & Nei M. Estimation of the number of nucleotide substitutions in the control region of mitochondrial DNA in humans and chimpanzees. Mol. Biol. Evol. 10, 512–526 (1993). 8336541 10.1093/oxfordjournals.molbev.a040023

[b29] Dwayne D. & Brown I. in Manual of Diagnostic Tests and Vaccines for Terrestrial Animals 2014, Chapter 2.3.4, Avian influenza. http://www.oie.int/fileadmin/Home/eng/Health_standards/tahm/2.03.04_AI.pdf (Accessed: 16th February2015).

