# Supplementary material for: Reassortant Highly Pathogenic Influenza A H5N2 Virus Containing Gene Segments Related to Eurasian H5N8 in British Columbia, Canada, 2014
Source: Sci Rep. 2015 Mar 25;5:9484. doi: 10.1038/srep09484 (PMC4372658; doi:10.1038/srep09484)
Supplement: Supplementary Information — Supplementary Material [file srep09484-s1.pdf]

Supplementary Material

Reassortant Highly Pathogenic Influenza A H5N2 Virus Containing Gene Segments Related to Eurasian H5N8 in British Columbia, Canada, 2014

John Pasick[1], Yohannes Berhane[1], Tomy Joseph[2], Victoria Bowes[2], Tamiko Hisanaga[1], Katherine Handel[1] & Soren Alexandersen[1*]

Phylogenetic analysis of the PB2 genes of HPAI H5N2 viruses isolated from Canadian poultry

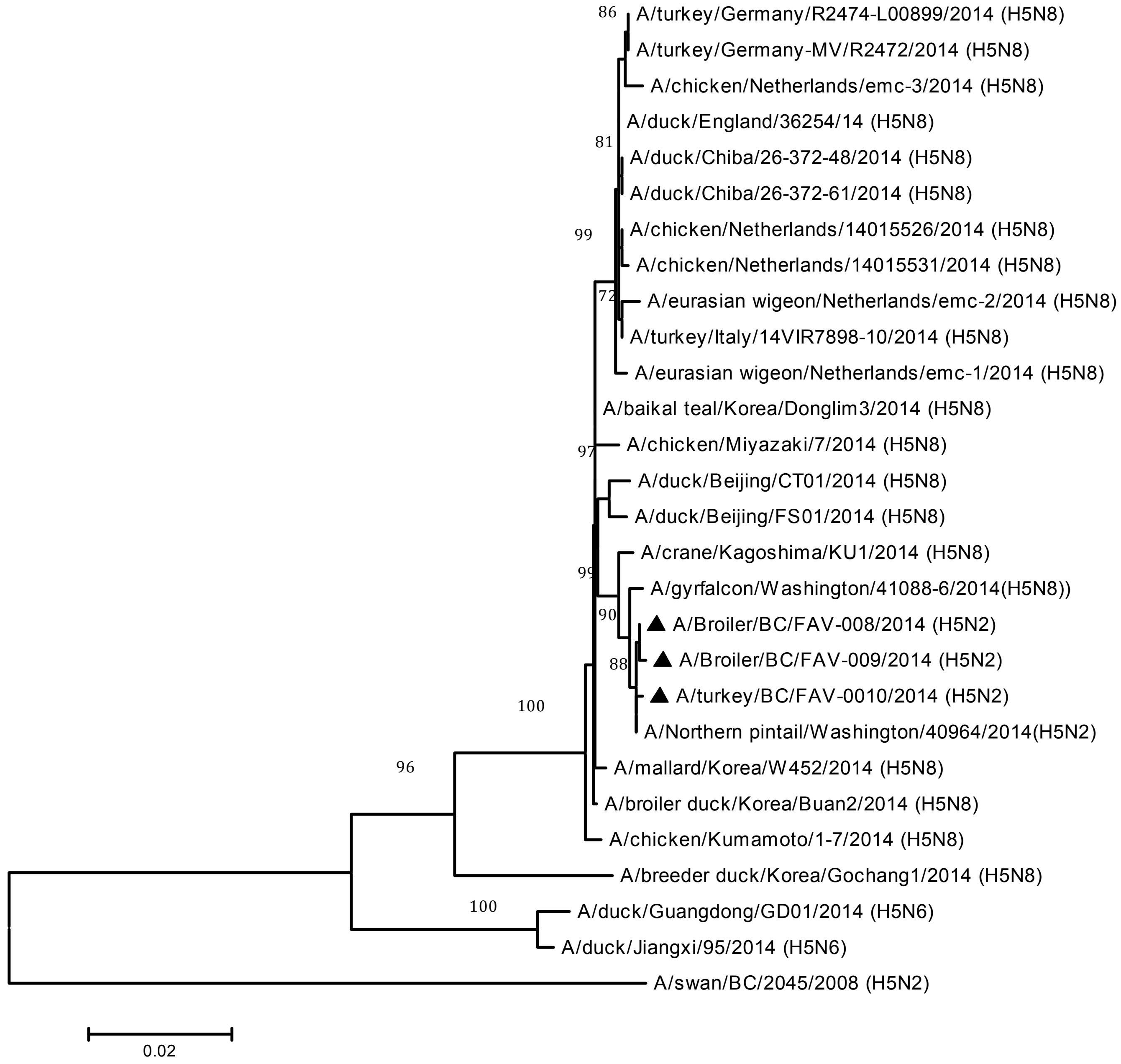

Phylogenetic analysis of the PB1 genes of HPAI H5N2 viruses isolated from Canadian poultry

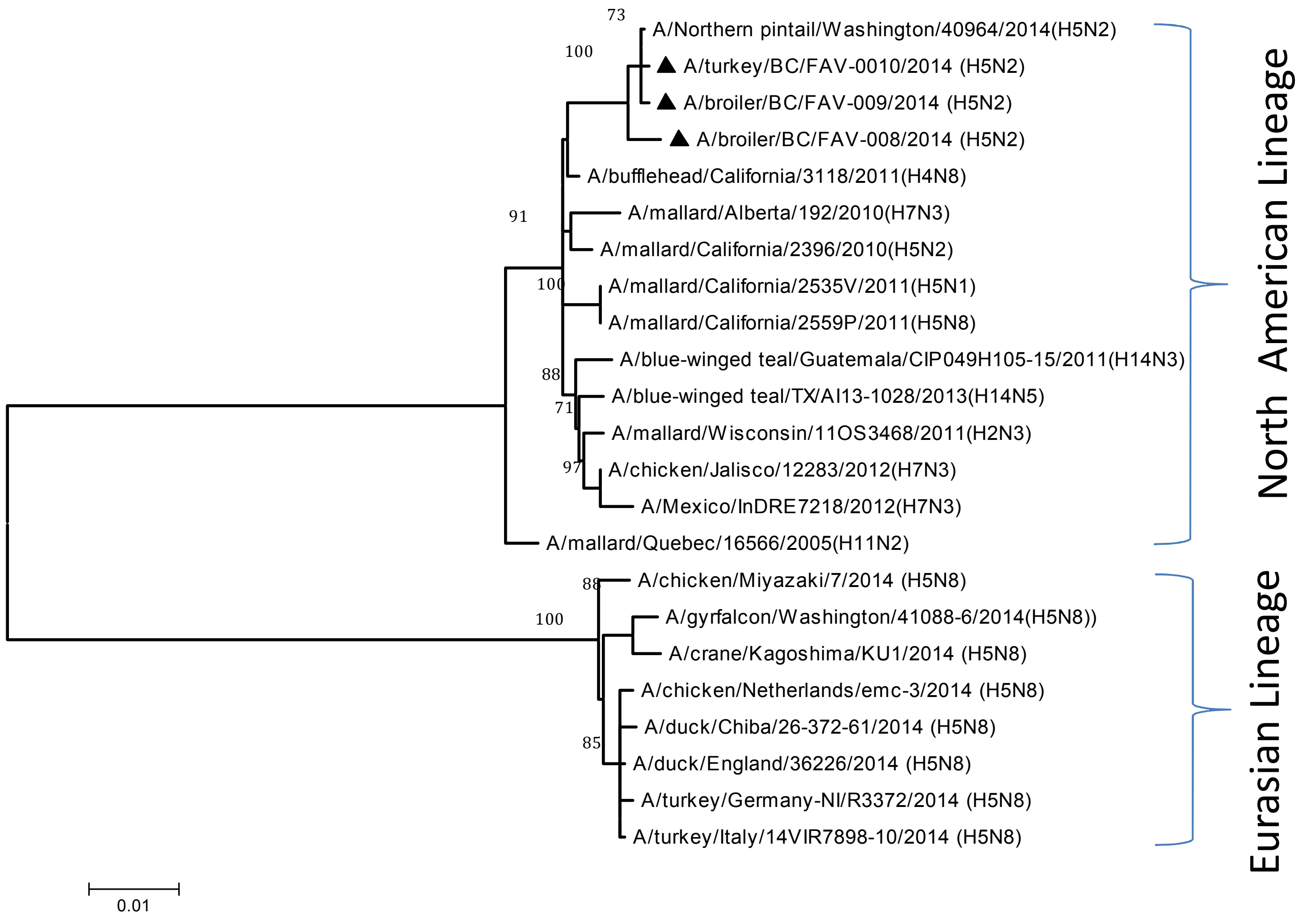

Phylogenetic analysis of the PA genes of HPAI H5N2 viruses isolated from Canadian poultry

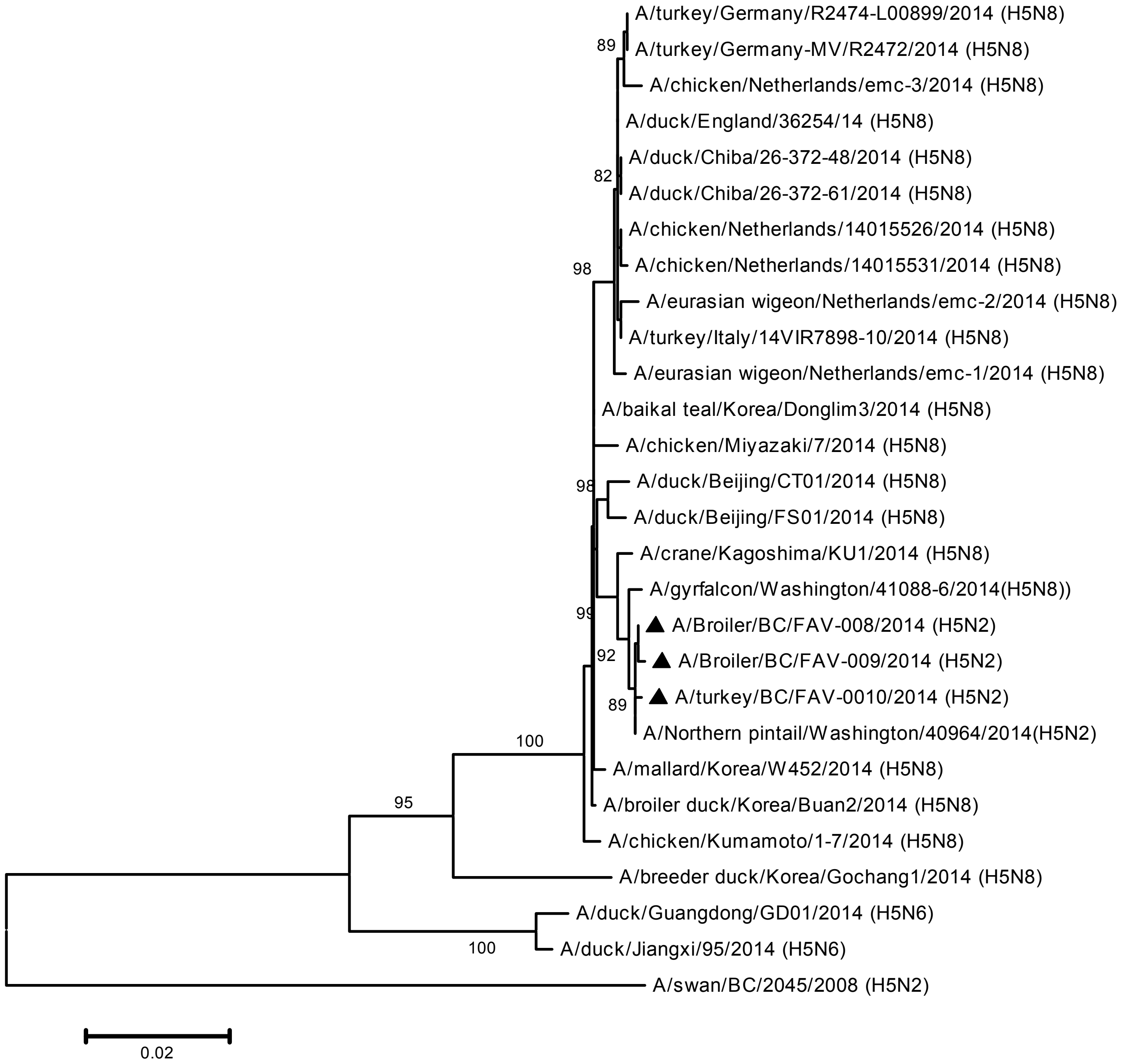

Phylogenetic analysis of the NP genes of HPAI H5N2 viruses isolated from Canadian poultry

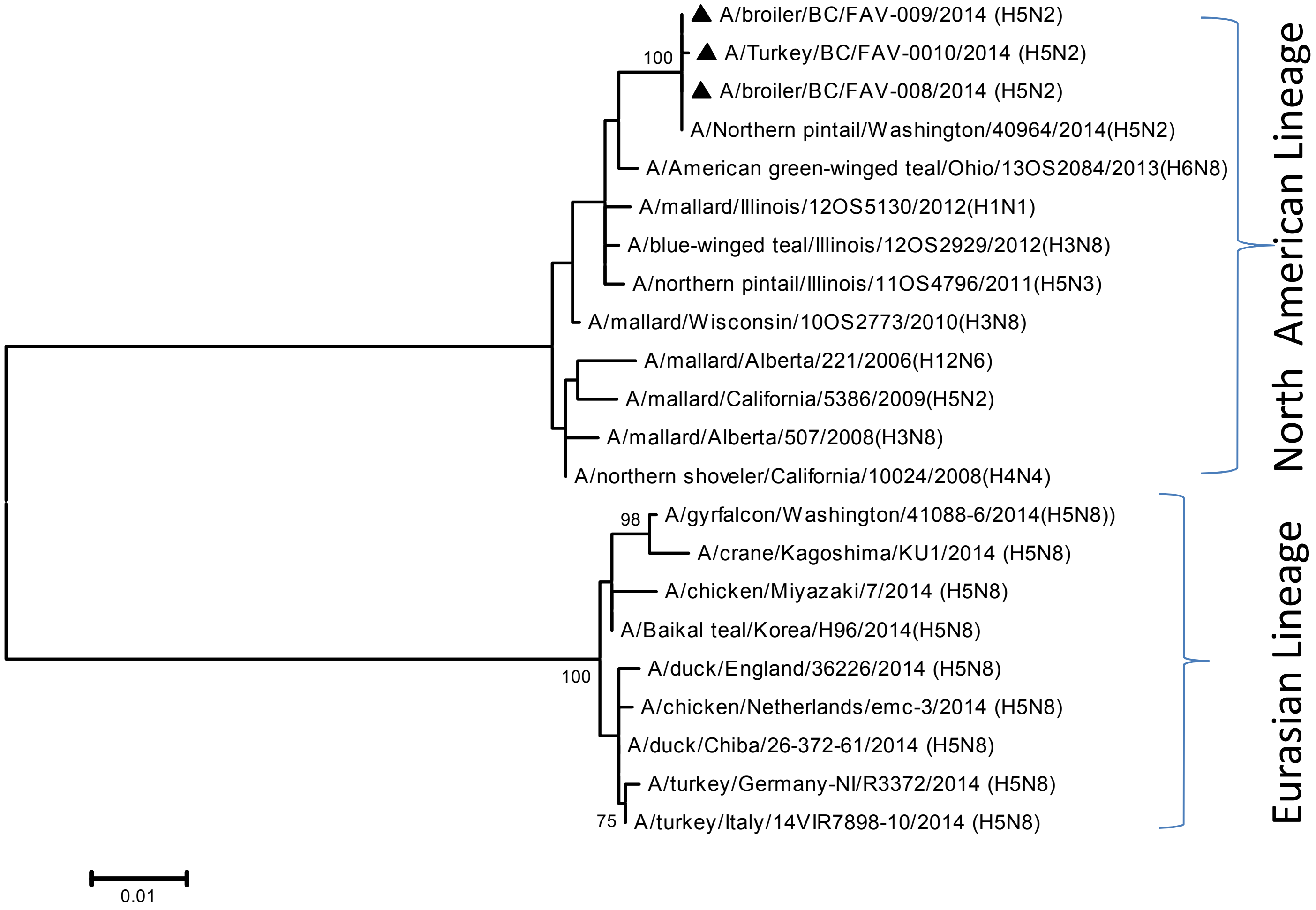

Phylogenetic analysis of the NA genes of HPAI H5N2 viruses isolated from Canadian poultry

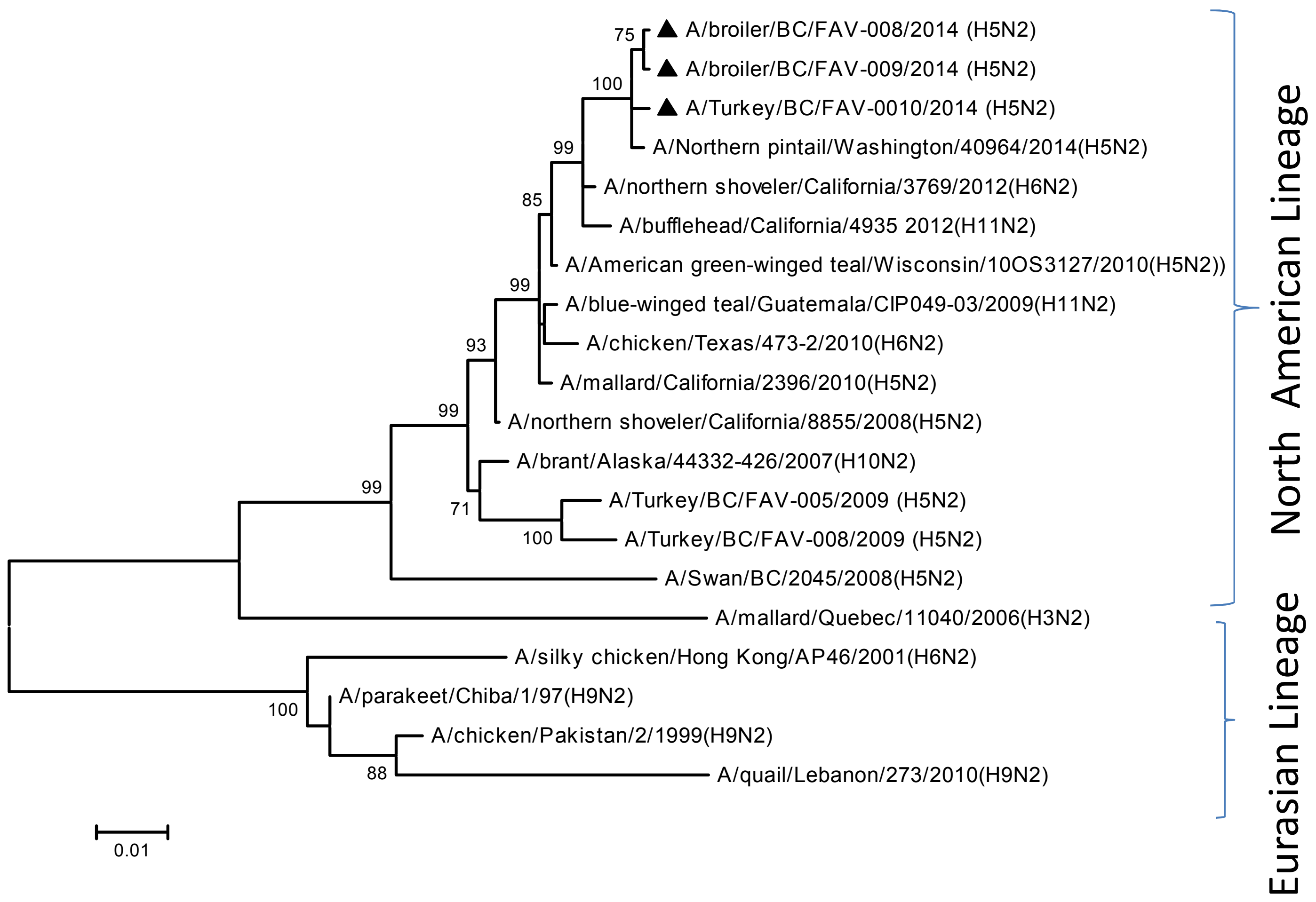

Phylogenetic analysis of the matrix genes of HPAI H5N2 viruses isolated from Canadian poultry

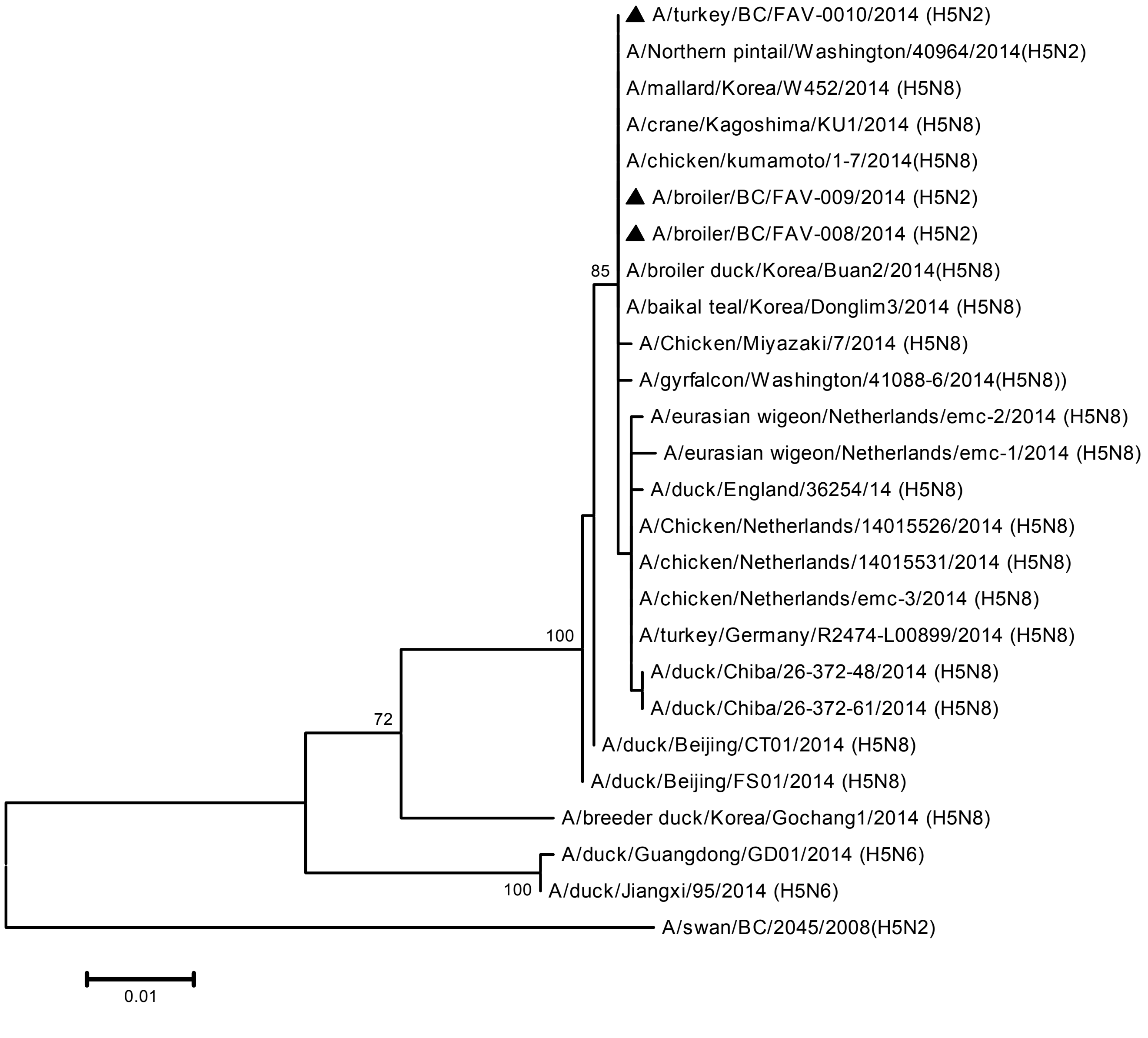

Phylogenetic analysis of the NS genes of HPAI H5N2 viruses isolated from Canadian poultry

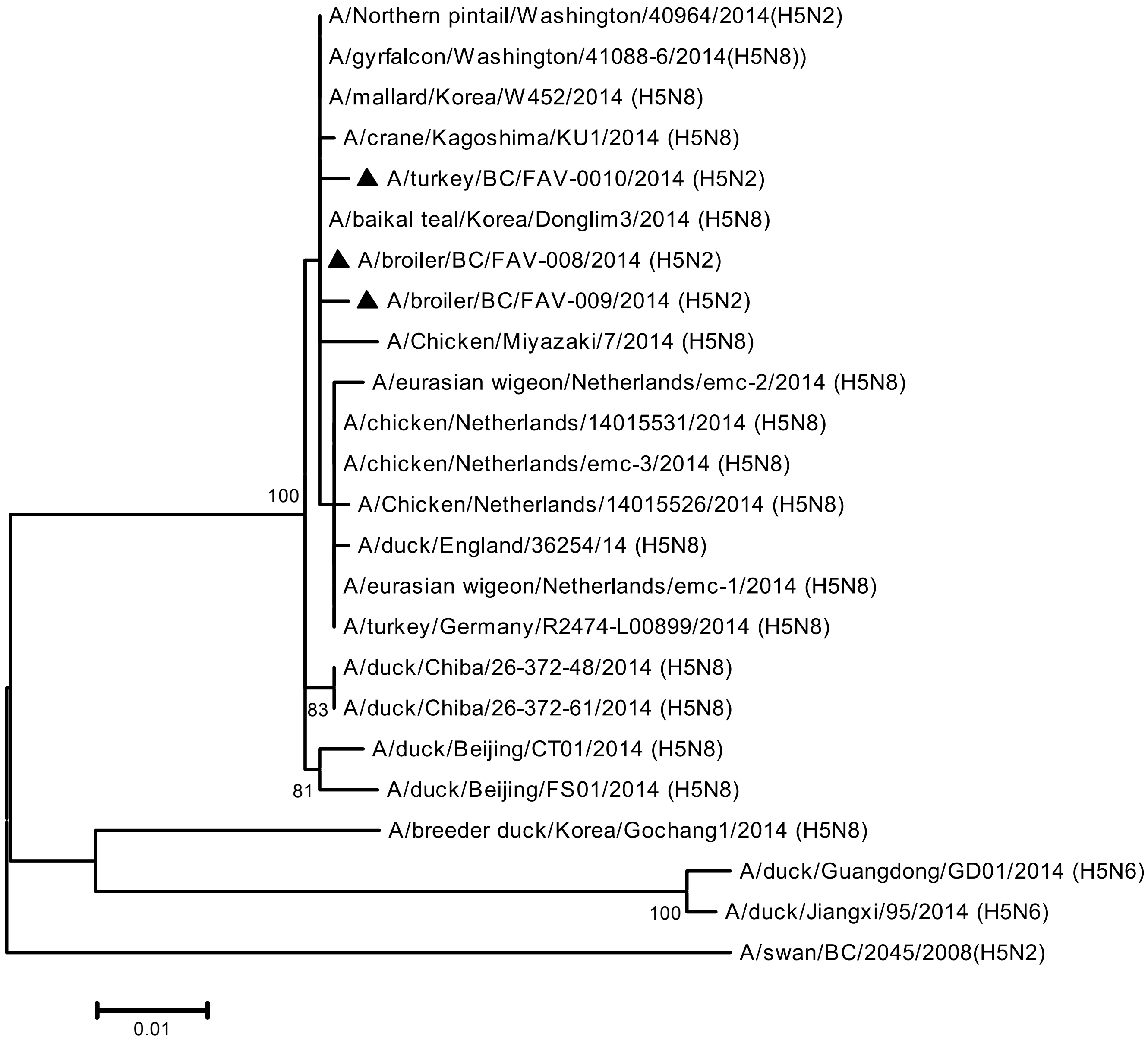

Supplementary Figure| Molecular Phylogenetic analysis of PB2, PB1, PA, NP, NA, M and NS gene segments of HPAI H5N2 viruses isolated from Canadian poultry.

The evolutionary history was inferred by using the Maximum Likelihood method based on the Tamura-Nei model. The tree with the highest log likelihood is shown. The percentage of trees in which the associated taxa clustered together is shown next to the branches. Initial tree(s) for the heuristic search were obtained by applying the Neighbor-Joining method to a matrix of pairwise distances estimated using the Maximum Composite Likelihood (MCL) approach. The tree is drawn to scale, with branch lengths measured in the number of substitutions per site. All positions containing gaps and missing data were eliminated. 500 bootstrap replicates were used to determine the reliability of the inferred trees with only bootstrap values above 70% shown. Evolutionary analyses were conducted in MEGA6.

Supplemental table 1: Acknowledgment of GISAID submitters

We acknowledge the authors, originating and submitting laboratories of the sequences from GISAID's EpiFlu ™ Database on which this research is based. The list is detailed below.
All submitters of data may be contacted directly via the GISAID website ***www.gisaid.org***

Isolate_Id	Isolate_Name	Location	Isolate_Submitter	Submitting_Lab	Authors	Originating_Lab
EPI_ISL_167140	A/turkey/Germany-MV/R2472/2014	Europe / Germany / Mecklenburg-Vorpommern	Elke Starick (Friedrich-Loeffler-Institut)	Friedrich-Loeffler-Institut		
EPI_ISL_169350	A/turkey/Italy/14VIR7898-10/2014	Europe / Italy / Veneto / Province of Rovigo	Alice Fusaro (Istituto Zooprofilattico Sperimentale Delle Venezie)	Istituto Zooprofilattico Sperimentale Delle Venezie	Luca,Tassoni; Silvia,Ormelli; Alessia,Schivo; Alice,Fusaro; Isabella,Monne; Giovanni,Cattoli	Istituto Zooprofilattico Sperimentale Delle Venezie
EPI_ISL_169390	A/crane/Kagoshima/KU1/2014	Asia / Japan / Kagoshima	Makoto Ozawa (Kagoshima University)	Kagoshima University		Kagoshima University
EPI_ISL_169424	A/chicken/Miyazaki/7/2014	Asia / Japan / Miyazaki	Takehiko Saito (National Institute of Animal Health)	National Institute of Animal Health		
EPI_ISL_159719	A/Chicken/Kumamoto/1-7/2014	Asia / Japan / Kumamoto	Michiyo Yoshizawa Harada (National Agriculture and Food Research Organization / National Institute of Animal Health)	National Agriculture and Food Research Organization	Kanehira,K.; Takemae,N.; Uchida,Y.; Tunekuni,R.; Hikono,H.; Saito,T.	National Institute of Animal Health
EPI_ISL_168075	A/chicken/Netherlands/14015531/2014	Europe / Netherlands / Provincie Utrecht / Gemeente Oudewater	Guus Koch (Central Veterinary Institute)	Central Veterinary Institute	Heutink, Rene; Harders, Frank; Verschuren-Pritz, Sylvia; Bossers, Alex; Koch, Guus; Bouwstra, Ruth	Central Veterinary Institute
EPI_ISL_167905	A/Chicken/Netherlands/14015526/2014	Europe / Netherlands / Provincie Utrecht / Gemeente Oudewater	Guus Koch (Central Veterinary Institute)	Central Veterinary Institute	Heutink, Rene; Harders, Frank; Verschuren-Pritz, Sylvia; Bossers, Alex; Koch, Guus; Bouwstra, Ruth	Central Veterinary Institute
EPI_ISL_168025	A/duck/Chiba/26-372-61/2014	Asia / Japan / Chiba	Takehiko Saito (National Institute of Animal Health)	National Institute of Animal Health		National Institute of Animal Health
EPI_ISL_168024	A/duck/Chiba/26-372-48/2014	Asia / Japan / Chiba	Takehiko Saito (National Institute of Animal Health)	National Institute of Animal Health		National Institute of Animal Health
EPI_ISL_167904	A/duck/England/36254/14	Europe / United Kingdom / East Riding of Yorkshire	Amanda Hanna (Animal and Plant Health Agency (APHA) / Virology)	Animal and Plant Health Agency (APHA)	Hanna, Amanda; Ellis, Richard; Ceeraz, Vanessa; Seekings, James; Londt, Brandon; Brookes, Sharon; Banks, Jill; Essen, Stephen; Brown, Ian	Animal and Plant Health Agency (APHA)
EPI_ISL_166694	A/duck/Beijing/CT01/2014	Asia / China / Beijing Municipality / Shunyi District	Di Liu (Institute of Microbiology)	Institute of Microbiology		Institute of Microbiology, Chinese Academy of Sciences
EPI_ISL_166693	A/duck/Beijing/FS01/2014	Asia / China / Beijing Municipality / Fangshan District	Di Liu (Institute of Microbiology)	Institute of Microbiology		Institute of Microbiology, Chinese Academy of Sciences
EPI_ISL_157610	A/broiler duck/Korea/Buan2/2014	Asia / Korea, Republic of	Initial import	Other Database Import	Lee,Y.-J.; Kang,H.-M.; Lee,E.-K.; Song,B.-M.; Jeong,J.; Kwon,Y.-K.; Kim,H.-R.; Lee,K.-J.; Hong,M.-S.; Jang,I.; Choi,K.-S.; Kim,J.-Y.; Lee,H.-J.; Kang,M.-S.; Jeong,O.-M.; Baek,J.-H.; Joo,Y.-S.; Park,Y.Ho.; Lee,H.-S.; Park,Y.H.	
EPI_ISL_157609	A/breeder duck/Korea/Gochang1/2014	Asia / Korea, Republic of	Initial import	Other Database Import	Lee,Y.-J.; Kang,H.-M.; Lee,E.-K.; Song,B.-M.; Jeong,J.; Kwon,Y.-K.; Kim,H.-R.; Lee,K.-J.; Hong,M.-S.; Jang,I.; Choi,K.-S.; Kim,J.-Y.; Lee,H.-J.; Kang,M.-S.; Jeong,O.-M.; Baek,J.-H.; Joo,Y.-S.; Park,Y.Ho.; Lee,H.-S.; Park,Y.H.	
EPI_ISL_171704	A/Baikal teal/Korea/H96/2014	Asia / Korea, Republic of	Initial import	Other Database Import	Jeong,J.; Kang,H.M.; Lee,E.K.; Song,B.M.; Kwon,Y.K.; Kim,H.R.; Choi,K.S.; Kim,J.Y.; Lee,H.J.; Moon,O.K.; Jeong,W.; Choi,J.; Baek,J.H.; Joo,Y.S.; Park,Y.H.; Lee,H.S.; Lee,Y.J.; Lee,Y.-J.; Kang,H.-M.; Lee,E.-K.; Song,B.-M.; Lee,K.-J.; Hong,M.-S.	
EPI_ISL_157611	A/baikal teal/Korea/Donglim3/2014	Asia / Korea, Republic of	Initial import	Other Database Import	Lee,Y.-J.; Kang,H.-M.; Lee,E.-K.; Song,B.-M.; Jeong,J.; Kwon,Y.-K.; Kim,H.-R.; Lee,K.-J.; Hong,M.-S.; Jang,I.; Choi,K.-S.; Kim,J.-Y.; Lee,H.-J.; Kang,M.-S.; Jeong,O.-M.; Baek,J.-H.; Joo,Y.-S.; Park,Y.Ho.; Lee,H.-S.; Park,Y.H.	
EPI_ISL_169281	A/eurasian wigeon/Netherlands/emc-2/2014	Europe / Netherlands / Provincie Utrecht / Gemeente Woerden / Between Kamerik and Kockengen	Josanne Verhagen (Erasmus Medical Center / Department of Virology)	Erasmus Medical Center	Fouchier, Ron A.M.; Verhagen, Josanne H.; Vuong, Oanh; Bestebroer, Theo; Van Vliet, Stefan; Van der Jeugd, Henk	Erasmus Medical Center
EPI_ISL_169280	A/eurasian wigeon/Netherlands/emc-1/2014	Europe / Netherlands / Provincie Utrecht / Gemeente Woerden / Between Kamerik and Kockengen	Josanne Verhagen (Erasmus Medical Center / Department of Virology)	Erasmus Medical Center	Fouchier, Ron A.M.; Verhagen, Josanne H.; Vuong, Oanh; Bestebroer, Theo; Van Vliet, Stefan; Van der Jeugd, Henk	Erasmus Medical Center
EPI_ISL_161972	A/duck/Jiangxi/95/2014		Guang Liu (BGI Shenzhen)	BGI Shenzhen	Bing,Xu;Tao,Zhang;Xiaowen,Li	
EPI_ISL_162467	A/duck/Guangdong/GD01/2014		Initial import	Other Database Import	Shen,H.; Xie,Q.	
EPI_ISL_139385	A/duck/Jiangsu/k1203/2010		Initial import	Other Database Import	Zhao,K.; Gu,M.; Zhong,L.; Duan,Z.; Zhang,Y.; Zhu,Y.; Zhao,G.; Zhao,M.; Chen,Z.; Hu,S.; Liu,W.; Liu,X.; Peng,D.	
EPI_ISL_156815	A/mallard duck/Shanghai/SH-9/2013	Fan Sheng Tao (Institute of Laboratory Animal Sciences, Chinese Academy)	Institute of Laboratory Animal Sciences, Chinese Academy		Fan,S.; Gao,X.;Ying,Y.; Guo,J.; Sun,W.; Wang,T.;Ren,Z.; Yu,Z.; Li,Y.; Zhao,Y.; Yang,S.;Gao,Y. ; Xia,X.	Institute of Military Veterinary, Academy of Military Medical Sciences
